# Ability to successfully use an epinephrine auto-injector after switching to a different device

**DOI:** 10.1186/2045-7022-5-S3-P5

**Published:** 2015-03-30

**Authors:** Robert Boyle, Annabella Procktor, Katherine Phillips, Camila Pinto, Heather Hanna, Thisanayagam Umasunthar

**Affiliations:** 1 Imperial College London, London, UK

## Rationale

Patients previously prescribed one epinephrine auto-injector device may be switched to an alternative device by their pharmacist or physician - sometimes without training on the new device. It is unclear whether “device switches” without retraining compromise the ability to deliver epinephrine.

## Methods

We evaluated mothers of food-allergic children participating in a UK study of epinephrine auto-injectors (EAI), 1 year after they were first trained to use an EAI, either Anapen or Epipen (old design). Participants' ability to deliver epinephrine using their device was assessed using a simulated anaphylaxis scenario. Participants then underwent repeat assessment using a different EAI device, randomly allocated, without training on the new device. The UK-approved EAIs Epipen (new/old designs), JEXT or Anapen were used, or Intelliject, an EAI with audio/visual prompts approved in North America as AuviQ(tm) and Allerject^TM^. ISRCTN29175528

## Results

We evaluated ability to deliver epinephrine in 108 participants. Overall success rates were similar using their original EAI 68/108 (63%) to the new device 65/108 (60%; P=0.775). However the outcome differed significantly for different types of device switch. Success rates were lower when switching between Anapen and either old Epipen, new Epipen or JEXT (6/18; 33%) compared with switching from old Epipen to either new Epipen or JEXT (30/42; 71%; P<0.009). Success rates were highest when switching from Anapen or old Epipen to Intelliject (26/28; 93%) compared with switching to other EAIs (39/80; 49%; P=0.000).

## Conclusions

The safety of EAI device switches varies according to the specific device. Switches to Intelliject appear to be safer than other forms of device switch.

